# Dopamine transporter forms stable dimers in the live cell plasma membrane in a phosphatidylinositol 4,5-bisphosphate–independent manner

**DOI:** 10.1074/jbc.RA118.006178

**Published:** 2019-01-31

**Authors:** Anand Kant Das, Oliver Kudlacek, Florian Baumgart, Kathrin Jaentsch, Thomas Stockner, Harald H. Sitte, Gerhard J. Schütz

**Affiliations:** From the ‡ Institute of Applied Physics, TU Wien, Getreidemarkt 9, A-1060, Vienna and; the § Center for Physiology and Pharmacology, Institute of Pharmacology, Medical University Vienna, Waehringerstrasse 13a, A-1090 Vienna, Austria

**Keywords:** membrane protein, dopamine transporter, single-molecule biophysics, dimerization, inositol phospholipid, protein–protein interaction, neurotransmitter, single-molecule brightness analysis, synaptic transmission

## Abstract

The human dopamine transporter (hDAT) regulates the level of the neurotransmitter dopamine (DA) in the synaptic cleft and recycles DA for storage in the presynaptic vesicular pool. Many neurotransmitter transporters exist as oligomers, but the physiological role of oligomerization remains unclear; for example, it has been speculated to be a prerequisite for amphetamine-induced release and protein trafficking. Previous studies point to an oligomeric quaternary structure of hDAT; however, the exact stoichiometry and the fraction of co-existing oligomeric states are not known. Here, we used single-molecule brightness analysis to quantify the degree of oligomerization of heterologously expressed hDAT fused to monomeric GFP (mGFP–hDAT) in Chinese hamster ovary (CHO) cells. We observed that monomers and dimers of mGFP–hDAT co-exist and that higher-order molecular complexes of mGFP–hDAT are absent at the plasma membrane. The mGFP–hDAT dimers were stable over several minutes, and the fraction of dimers was independent of the mGFP–hDAT surface density. Furthermore, neither oxidation nor depletion of cholesterol had any effect on the fraction of dimers. Unlike for the human serotonin transporter (hSERT), in which direct binding of phosphatidylinositol 4,5-bisphosphate (PIP_2_) stabilized the oligomers, the stability of mGFP–hDAT dimers was PIP_2_ independent.

## Introduction

The human dopamine transporter (hDAT)^3^ is a presynaptic protein containing 12 transmembrane helices (TMHs). It is involved in dopamine (DA) homeostasis by high-affinity reuptake of dopamine from the synaptic cleft (1, 2) thereby terminating chemical signal transduction in dopaminergic synapses. hDAT belongs to the Na^+^- and Cl^−^-dependent solute carrier 6 (SLC6) family (3, 4), which also includes the transporters for serotonin (SERT), norepinephrine (NET), γ-amino acid transporters (GAT), glycine (GlyT), and several other amino acids and osmolytes (4, 5). hDAT is a primary target for illicit drugs and psychostimulants such as cocaine and amphetamines (6, 7). Alterations in hDAT functions could lead to diseases including depression, attention deficit hyperactivity disorder, bipolar disorders, schizophrenia, or Parkinson's disease (8).

Over the last few decades, oligomerization of proteins has been an intense area of research from the perspective of protein evolution and opportunities for functional control. Oligomerization has been proposed to play a critical role in the regulation of cellular function including signal transduction and immune response (9, 10) as it may offer stability, higher order complexity, and compartmentalization of reactions (11). Oligomers could also have deleterious effects on cells as observed in the case of neurodegenerative disorders (12–14). For a large number of proteins, there are specific binding domains that facilitate or modulate oligomerization (15, 16). Lipids have also been found to be important in membrane protein folding and oligomerization (17–19). For example, we recently found a prominent role of the minor phospholipid phosphatidylinositol 4,5-bisphosphate (PIP_2_) for human SERT (hSERT) oligomerization and function (20, 21).

Like several members of the SLC6 family that were shown to form oligomers (22–27), including our recent studies on hSERT (20, 28), hDAT has also been reported to exhibit oligomeric quaternary organization as observed in radiation inactivation studies (29, 30), co-purification experiments with differentially tagged epitopes (31, 32), co-immunoprecipitation and fluorescence resonance energy transfer (FRET) microscopy studies in heterologous expression systems (33) and transfected neurons (34). In addition, cross-linking experiments suggest the involvement of TMHs 4 and 6 in the interfaces of symmetrical hDAT dimers (31, 35, 36). More recently, unbiased molecular dynamics simulations followed the dimerization of two randomly oriented hDAT monomers and reported the existence of a limited number of symmetric and asymmetric dimers (16). Additionally, the bundle domain comprising of TMHs 1, 2, 6, and 7 was largely excluded from the dimer interface, whereas the scaffold domain formed a significant part of the dimer interface (16).

Interestingly, the exact functional significance of such oligomerization behavior is largely unknown and has been widely speculated (22, 37). Oligomerization of SLC6 family members has been proposed to be important for the following processes. (i) As a quality control checkpoint before trafficking of correctly folded proteins from the endoplasmic reticulum (ER) and/or the Golgi to the cell surface (22, 38). It has been observed that oligomerization-deficient mutants of GAT1 are retained in the ER, but they remain capable of transporting γ-amino butyric acid (GABA) in vesicular preparations (39). Interestingly, the oligomeric state does not seem to influence the neurotransmitter uptake activity, as observed in these GAT1 mutants (39). Inversely, oligomerization of both SERT and GAT1 is not influenced by various transporter substrates and inhibitors (23). In the case of hDAT, it has been observed that formation of the oligomeric complex is essential for correct trafficking of the transporter complex to the cell membrane (31, 33, 40–43). (ii) Transporter oligomerization has been suggested to entail functional consequences (25, 44, 45) and to support the countertransport model of neurotransmitter transport (37), where amphetamine binds to one subunit of an SLC6 oligomer and the transporter-mediated efflux of neurotransmitter is subsequently performed by the neighboring subunit (46). In this model, only SLC6 complexes having at least two transporter subunits would be susceptible to amphetamine-elicited neurotransmitter efflux.

The role of membrane cholesterol in modulating hDAT function has been extensively studied (47–50). Depletion of membrane cholesterol was shown to lower the affinity of hDAT for DA and its uptake velocity in [^3^H]DA uptake assays (47, 48) and efflux rate (51). Although these studies support the oligomerization behavior of hDAT, they could not quantify the exact subunit stoichiometry, direct modulation by cholesterol–hDAT interaction, and the fraction of different co-existing oligomeric states in live cell membrane, their interaction kinetics, and factors influencing its oligomerization behavior.

In this study, we used single-molecule brightness analysis to probe the oligomerization behavior of hDAT fused to monomeric GFP (mGFP–hDAT) in the plasma membrane of living Chinese hamster ovary (CHO) cells. In the case of GFP it is known that *n*-mer complexes give rise to *n*-fold increased mean brightness compared with monomer constructs (52). The according brightness probability distributions of *n*-mers can be calculated from the GFP monomer signals (53, 54). Brightness analysis was successfully applied for characterizing, *e.g.* the oligomeric state of an ion channel (53), and the serotonin transporter (20, 28). Here, we observed that mGFP–hDAT in the plasma membrane predominantly showed the co-existence of monomers and dimers that were found to be independent of hDAT surface density. Furthermore, perturbation of membrane cholesterol either by oxidation or depletion had no impact on the oligomeric distribution of hDAT. Using a modified photobleaching protocol (28), we found that the mGFP–hDAT dimers were stable at least on a time scale of several minutes. In contrast to hSERT, where direct binding of PIP_2_-mediated stable oligomer formation (20), we obtained evidence that perturbation of PIP_2_ levels did not have any impact on hDAT dimerization.

## Results

### DAT co-exists as monomers and dimers in the live cell plasma membrane

**Figure 1.**
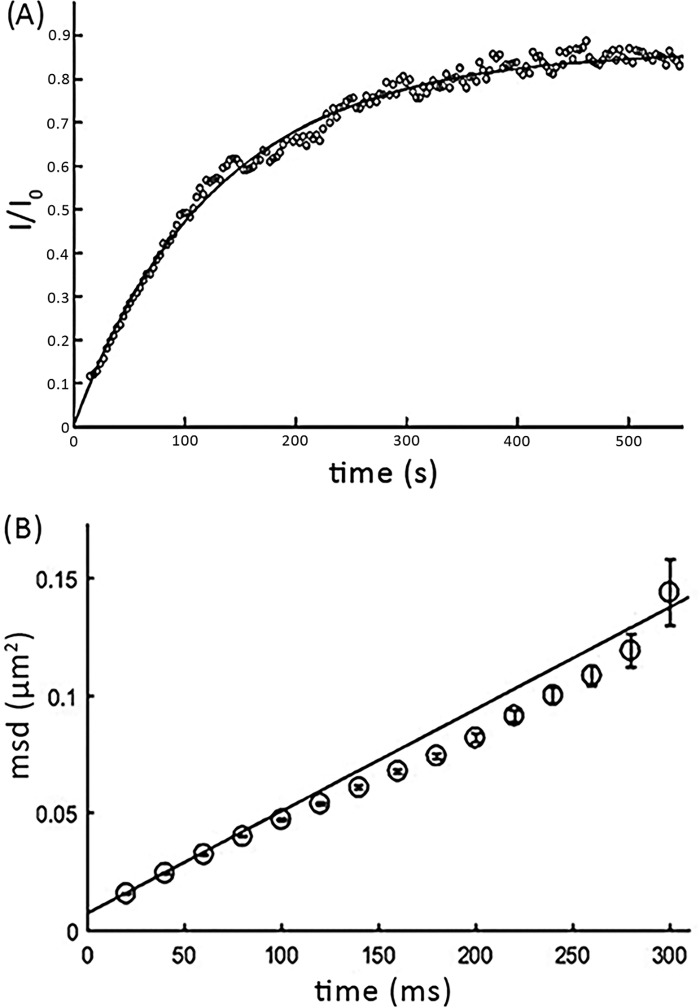
**Mobility of mGFP–hDAT at the live cell plasma membrane.** *A,* to determine the mobile fraction of mGFP–hDAT, FRAP experiments were performed on cells expressing mGFP–hDAT. The integrated intensity of the bleached area (*I*) was normalized to the fluorescence intensity before bleaching (*I*_0_) and plotted over time (*black circles*, showing a representative recovery curve). The average over 11 cells yielded a mean mobile fraction of m = 78 ± 10%. *B,* using single molecule tracking, the diffusion coefficient *D* was determined. Mean square displacements were calculated for a range of time lags (*t*-lag) and *D* was determined from the first two points in the MSD plot according to *MSD* = 4*Dt_lag_* + *offset*, yielding *D* = 0.118 ± 0.002 μm^2^/s. *Error bars* show the mean ± S.E.

We determined the subunit stoichiometry of mGFP–hDAT by heterologously expressing the construct as a stable mGFP–hDAT CHO cell line. Because it was shown earlier that multiple molecular determinants in the carboxyl terminus of hDAT regulate its export from the ER (42), we fused mGFP to the cytosolic N terminus of hDAT. The fluorescence signal was recorded from the plasma membrane, which is close to the glass surface, by using total internal reflection fluorescence (TIRF) microscopy. The transporter showed considerable mobility: fluorescence recovery after photobleaching (FRAP) yielded a mobile fraction of 78 ± 10% (Fig. 1*A*), and single-molecule tracking showed a lateral mobility of D = 0.118 ± 0.002 μm^2^/s (Fig. 1*B*). mGFP–hDAT was functional as indicated from [^3^H]DA uptake measurements in cells transiently transfected with mGFP–hDAT (Fig. S1). N-terminal mGFP fusion to hDAT does not have functional consequences on DA transport, as WT hDAT and mGFP–hDAT showed comparable *K_m_* values, 2.98 ± 0.38 μm for hDAT and 4.11 ± 0.85 μm for mGFP–hDAT. The difference in the *V*_max_ arises from lower surface expression of the mGFP–DAT construct compared with the WT construct.

**Figure 2.**
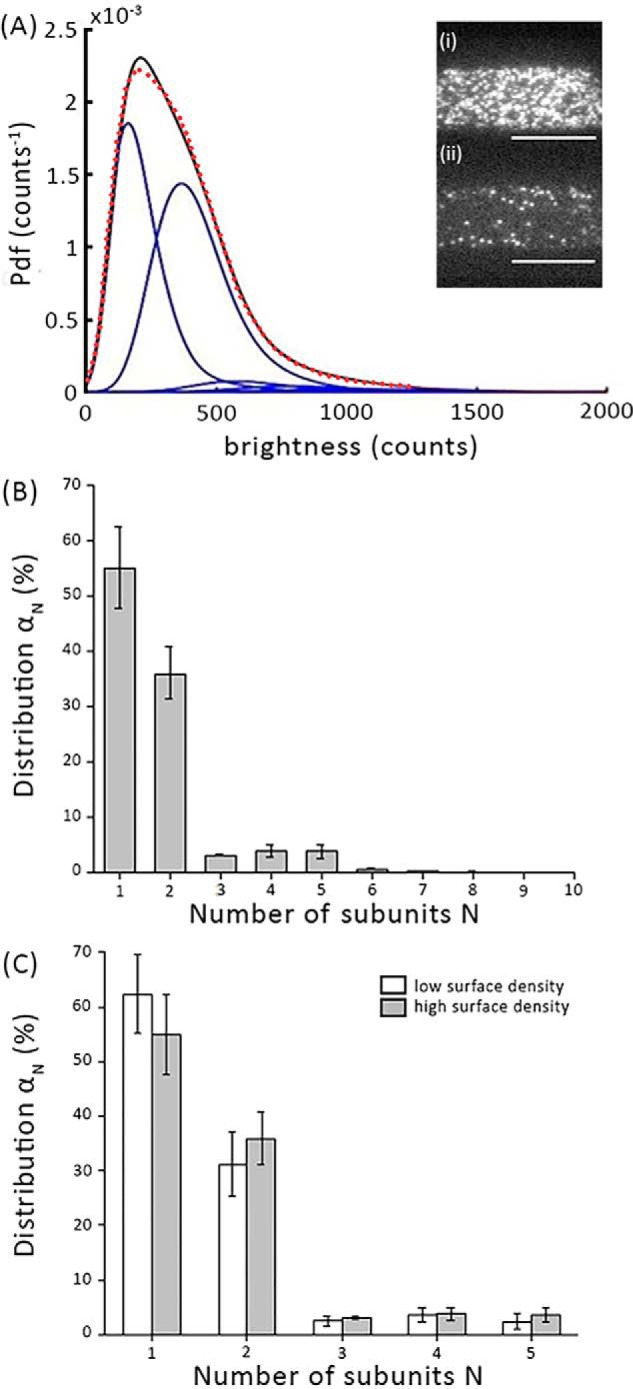
**Determination of mGFP–hDAT oligomer distribution on the plasma membrane using single molecule brightness analysis.** *A,* the obtained brightness distribution of the oligomeric fractions are plotted as probability density function (*Pdf*) (*black line*): the fit is shown as a *dashed red line* and the *n*-mer contributions as *blue lines. Inset,* representative pre-bleach (*i*) and post-bleach (*ii*) fluorescence images are shown from a region of the plasma membrane. *Scale bar*, 10 μm. *B,* the distribution of oligomeric states (*gray bars*) of mGFP–hDAT shows the presence of monomers (∼55%) and dimers (∼35%) co-existing at the plasma membrane (*n* = 103 cells). *C,* the oligomeric distribution is independent of the density of mGFP–hDAT at the plasma membrane when evaluated at a mean density of ∼5 mGFP–hDAT/μm^2^ for low surface density and ∼40 mGFP–hDAT/μm^2^ for high surface density (*n* = 90 cells). *Error bars* show the mean ± S.E.

At typical expression levels, fluorescently labeled membrane proteins are present at high surface density, so that single-molecule signals cannot be separated. We hence made use of a method termed “Thinning out clusters while conserving stoichiometry of labeling” (TOCCSL) (54). In conjunction with single molecule brightness analysis, TOCCSL allows for the quantification of the oligomeric state of fluorescently labeled mobile membrane constituents. After recording a pre-bleach image for control, a distinct small area of the cell membrane is irreversibly photobleached. The photobleached region is confined by imaging an aperture onto the sample. Ideally, the protein complexes are either entirely photobleached (inside the analysis region) or remain entirely fluorescent (outside the analysis region), which can be assessed by a control image recorded immediately after the bleaching pulse. Due to Brownian motion, unbleached molecules diffuse into the bleached analysis region. TOCCSL makes use of the very onset of the recovery process: individual molecules or clusters diffuse into the bleached area and can be resolved as single, clearly distinguishable fluorescent spots. Fig. 2*A* shows the brightness distribution obtained in the TOCCSL images where the brightness distribution is plotted as a probability density function depicted in *black*, whereas the fit and the *n*-mer contributions are shown in *red* and *blue*, respectively. Fig. 2*B* reveals the oligomeric distribution of mGFP–hDAT as obtained by our single-molecule brightness analysis. Interestingly, we observe only the presence of monomers (∼55%) and dimers (∼35%) co-existing in the live cell plasma membrane. Marginal fractions of higher order aggregates are below the detection sensitivity of TOCCSL (55).

Next, we were interested to probe whether the process of dimer formation equilibrates at the plasma membrane, as observed for the oligomerization of G protein–coupled receptors (56). In this case, the higher surface density of mGFP–hDAT would drive the equilibrium toward higher oligomeric structures and vice versa. For this, we separately analyzed data from cells showing either low (∼5 molecules of mGFP–hDAT/μm^2^) or high (∼40 molecules of mGFP–hDAT/μm^2^) expression levels. Although the two groups differed by a factor of eight in their surface densities, we did not observe a substantial difference in their oligomeric size (Fig. 2*C*). Unfortunately, for technical reasons it was not possible to analyze cells with higher expression levels of mGFP–hDAT because of intracellular retention of hDAT, leading to strong background signals that prevented single molecule imaging. Our data suggests that mGFP–hDAT oligomer association and dissociation is not a continuous process at the plasma membrane.

### mGFP–hDAT forms stable dimers

**Figure 3.**
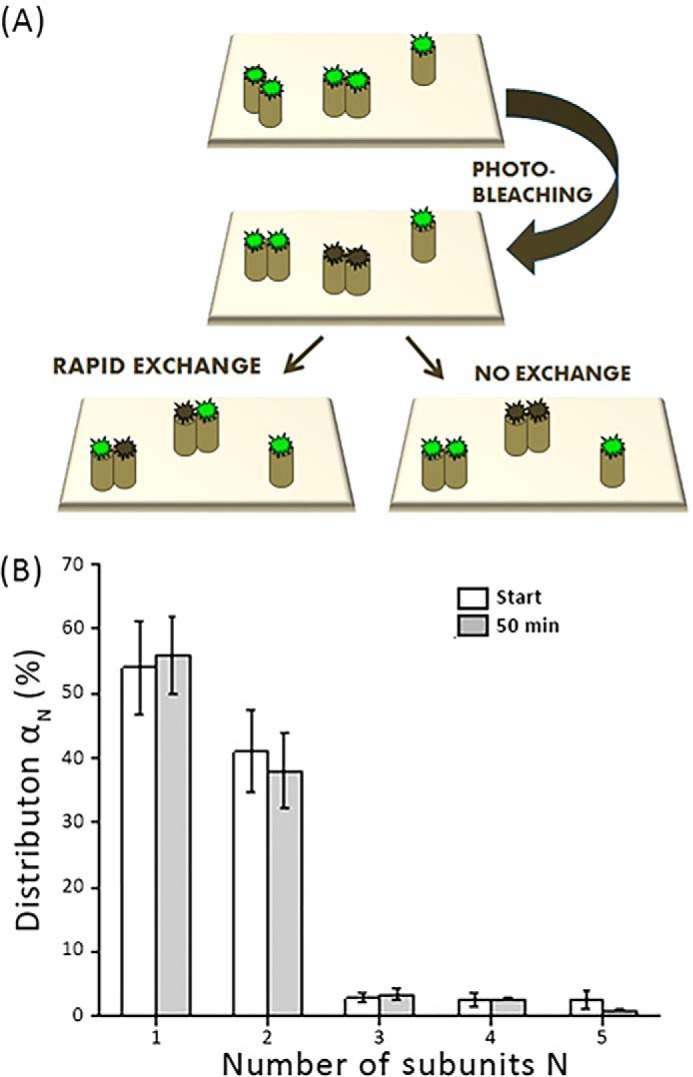
**To evaluate the stability of the mGFP–hDAT dimers.** *A,* the transporter complexes in a cell were photobleached using a sequence of 10 TOCCSL experiments, and the oligomeric distribution was monitored for 50 min. *A,* the sketch shows two potential cases: transient interaction of mGFP–hDAT would lead to a rearrangement after photobleaching, resulting in a mixed population of bleached (*gray*) and unbleached (*green*) molecules per dimer; in this case, the number of unbleached dyes per dimer would be reduced (*left*). In contrast, stable interaction would produce either completely bleached or unbleached dimers, without altering the distribution (*right*). *B,* dimeric distribution α_N_ at the start of the experiment (*white bars*), and after 10 consecutive TOCCSL experiments performed every 5 min (*gray bars*) (*n* = 37 cells). No change in the dimeric distribution was observed. *Error bars* show the mean ± S.E.

To discriminate between rapid subunit exchange and stable association, we have earlier designed a modified protocol based on repetitive TOCCSL runs on the very same cells (28). Here, the TOCCSL run is repeated every 5 min for 50 min on the same cell. After each TOCCSL run, the oligomeric distribution is monitored on the recovery image using single-molecule brightness analysis. Pooling the data obtained from multiple cells provides brightness distributions as a function of time. Two scenarios can be discriminated: if the interaction of subunits within a given complex was stable, repetitive TOCCSL runs would reduce the total number of observed fluorescent spots on the plasma membrane, but would not alter the brightness distribution. In contrast, if the exchange rate of subunits between dimers was high, bleached subunits from the previous TOCCSL experiments would rearrange with unbleached subunits. Over time, this mixing would increase the number of complexes comprised of both dark and fluorescent subunits, thereby shifting the apparent distribution toward monomers (Fig. 3*A*). At the plasma membrane, we observed massive depletion of fluorescent molecules after 10 repeats (*i.e.* after 50 min) (Fig. S2), whereas the mGFP–hDAT dimer fraction in subsequent TOCCSL runs (Fig. 3*B*) remained unchanged indicating that no exchange of dimer subunits took place within the 50-min time scale. We therefore infer that the dimers are stable at least over several minutes at the plasma membrane.

### Oxidation or depletion of plasma membrane cholesterol does not affect the dimer distribution

**Figure 4.**
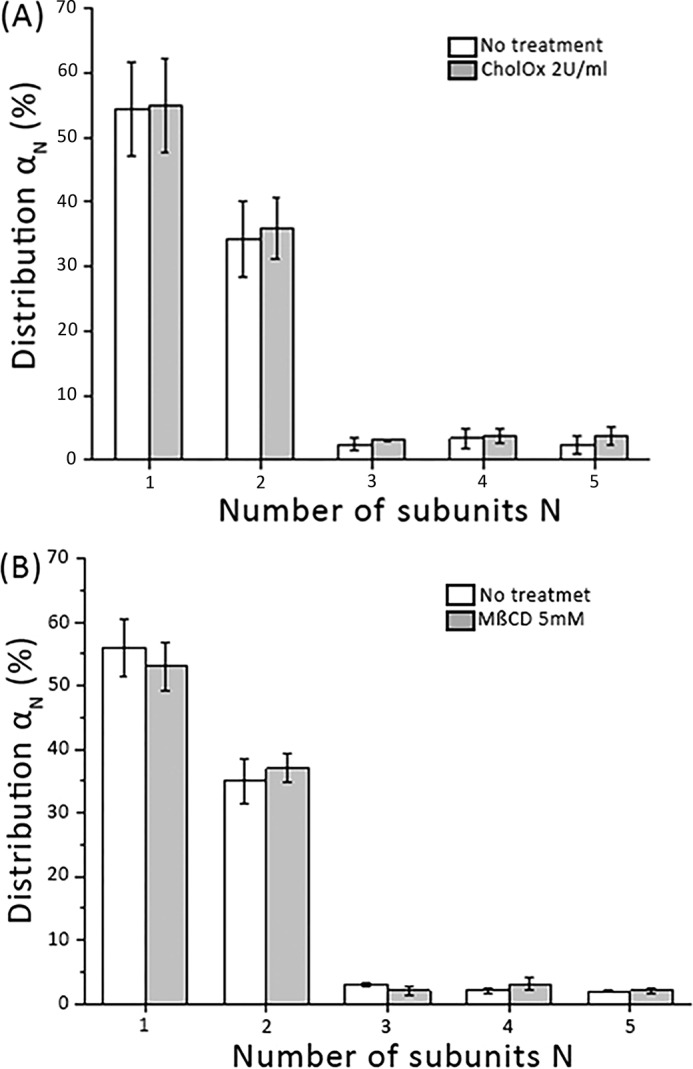
**mGFP–hDAT dimeric distribution is not affected by perturbation of cholesterol.** *A,* comparison of the dimeric mGFP–hDAT distributions in the unaltered lipid environment of the plasma membrane (*white bars*) and after cholesterol oxidation with cholesterol oxidase (2 units/ml for 30 min). No apparent influence of the cholesterol content is observable (*n* = 61 cells). *B,* cholesterol depletion by MβCD (5 mm for 1 h) does not affect the dimer distribution (*gray bars*) when compared with the unaltered lipid environment of the plasma membrane (*white bars*) (*n* = 43 cells). *Error bars* show the mean S.E.

It has been reported earlier that depletion of membrane cholesterol reduced both DA uptake and efflux rates (51). We hence studied the effect of cholesterol on the dimer distribution of mGFP–hDAT. Cholesterol oxidase oxidizes the hydroxyl group of membrane cholesterol to a keto group (57, 58). When we perturbed membrane cholesterol with the cholesterol oxidase (2 units/ml for 30 min) we found no effect on the dimer fraction (∼35%) of mGFP–hDAT (Fig. 4*A*). This was found to be independent of plasma membrane expression of mGFP–hDAT (Fig. S3).

In an alternative approach, we depleted cholesterol using methyl-β-cyclodextrin (MβCD) (59) (5 mm for 1 h), however, we found that the dimer fraction remained unchanged when compared with untreated cells (Fig. 4*B*), which was again found to be independent of the density of mGFP–hDAT on the cell surface (Fig. S4). These data show that perturbing the membrane cholesterol concentration either by oxidation or by depletion has no effect on the dimer distribution of mGFP–hDAT.

### Stability of the mGFP–hDAT dimers does not depend on PIP_2_ availability

**Figure 5.**
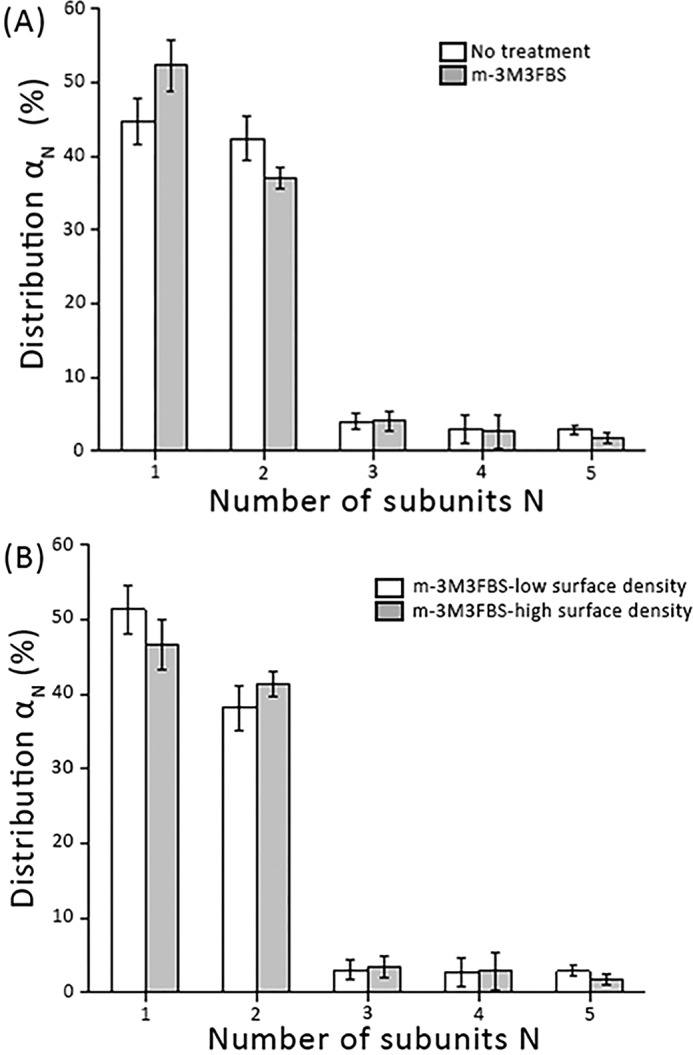
**mGFP–hDAT dimerization at the plasma membrane is independent of PIP_2_ levels.** *A,* we enzymatically depleted PIP_2_ at the plasma membrane via activation of PLCγ by incubating cells for 20 min with the direct PLCγ activator m-3M3FBS (25 μm). m-3M3FBS did not yield any effect on the oligomeric distribution (*gray bars*) compared with the untreated cells (*white bars*) (*n* > 80 cells per experimental condition). mGFP–hDAT surface densities were similar: 35 ± 4 μm^2^ (*white bars*) and 33 ± 5 μm^2^ (*gray bars*). *B,* the oligomeric distribution is plotted for various mGFP–hDAT surface density (*n* > 40 cells) following PIP_2_ depletion via m-3M3FBS; mGFP–hDAT densities are ∼7 mGFP–hDAT/μm^2^ for low surface density and ∼40 mGFP–hDAT/μm^2^ for high surface density. *Error bars* show the mean S.E.

It has been observed that the hDAT N terminus interacts electrostatically with PIP_2_ and that this interaction impairs DA efflux, but not uptake (60). We hence probed whether PIP_2_ contributes to the stability of mGFP–hDAT dimers. PLCγ is a highly specific enzyme for PIP_2_ hydrolysis, leaving other phosphoinositides virtually unaffected (61). We used the direct PLCγ-activator 2,4,6-trimethyl-*N*-(3-trifluoromethylphenyl)benzenesulfonamide (m-3M3FBS) (25 μm) to enzymatically deplete PIP_2_ at the plasma membrane. Fig. 5*A* shows that PIP_2_ depletion had no influence on the dimer distribution of mGFP–hDAT. The mean surface density of the transporter was not affected upon treatment with m-3M3FBS (Fig. S5). The lack of effect was consistent among all tested surface densities of mGFP–hDAT (Fig. 5*B*). Taken together, mGFP–hDAT forms stable dimers at the plasma membrane in a PIP_2_-independent manner.

## Discussion

The physiological role of hDAT is to maintain DA homeostasis in the synaptic nerve terminals by rapid reuptake of DA from the synaptic cleft for subsequent storage and release (62). Oligomerization is a phenomenon observed for a significant fraction of cellular proteins (11) including members of the monoamine transporter family (22). It is generally assumed that oligomerization of transporters of the SLC6 family plays a role in such diverse processes as membrane trafficking, transporter function, regulation, and substrate turnover (63). Concerning trafficking, protein oligomerization serves as a quality control mechanism to enable the export from the ER and/or the Golgi apparatus to the plasma membrane (38). Concerning function, transporter oligomerization has been suggested to be of importance for forward transport (25, 44, 45) and to establish the basis for the countertransport model proposed for transporter-mediated efflux of neurotransmitters (37, 46). In support of this latter model, superresolution microscopy revealed the presence of hDAT nanodomains in the plasma membrane (64). This could have functional implications such as regulation of transporter internalization (50) and amphetamine-induced dopamine efflux (47, 65).

Here, we examined the homo-oligomerization behavior of mGFP–hDAT in the plasma membrane of CHO cells by determining the oligomeric distribution and the interaction kinetics of the protomers using single-molecule brightness analysis. The mGFP was fused to the N terminus instead of the C terminus as it is critical for surface expression of hDAT (22, 66–68) and is vital for hDAT recognition by SEC24 as part of the ER export machinery (68, 69). We tried to compare and contrast the oligomerization behavior of hDAT with our previous findings with the hSERT to comprehend the nature of molecular association of members of the SLC6 family. Because the TOCCSL illumination protocol is blind to the immobile fraction, all our observations pertaining to the single-molecule analysis comprise only the mobile fraction which represents ∼80% of hDAT molecules at the plasma membrane (Fig. 1*A*). Surprisingly, we observed that mGFP–hDAT formed only monomers (∼55%) and dimers (∼35%), whereas higher order oligomers were absent (Fig. 2*B*). This is different to our previous observations for hSERT, where significant fractions of higher oligomeric structures of at least up to pentamers were observed in the plasma membrane (28).

A modified bleaching protocol in conjunction with the brightness analysis (referred in the study as repeat TOCCSL) allowed us to study the exchange kinetics of subunits within the multimers. Our group had earlier observed that mGFP–hSERT oligomers exhibited a dynamic equilibrium at the ER membrane due to its ability to rapidly exchange subunits within oligomers, which became kinetically trapped after trafficking to the plasma membrane, indicating a spatial decoupling of the oligomerization process at various sites within the cell (20). At the plasma membrane, the degree of oligomerization was found to be independent of hSERT surface density (28). Similar to what was observed earlier with hSERT, the distribution of mGFP–hDAT dimers is insusceptible to expression levels of mGFP–hDAT (Fig. 2*C*) indicating that formation and dissociation of mGFP–hDAT dimers does not occur at the plasma membrane. Consistently, the repeated TOCCSL protocol to determine the stability of mGFP–hDAT dimers showed that they are stable just like hSERT oligomers at least for a time window of 50 min (Fig. 3*B*). Taken together, the results indicate that the dimers behave similar to the hSERT oligomers in terms of stability.

Earlier it was reported that partitioning of hSERT and hDAT into lipid microdomains modulates its uptake activity (48, 70). We wanted to probe whether the perturbation of cholesterol at the plasma membrane had any influence on the dimeric distribution of mGFP–hDAT. Cholesterol oxidase catalyzes the oxidation of cholesterol resulting in the formation of cholestenone (cholest-4-en-3-one) (57), whereas methyl-β-cyclodextrin depletes membrane cholesterol (59). Currently, this is regarded as the canonical test for the involvement of plasma membrane rafts for the interaction process (71). We observed that the dimer fraction of mGFP–hDAT is affected neither by the action of cholesterol oxidase (Fig. 4*A,* Fig. S3) nor by cholesterol depletion from the cell surface using MβCD (Fig. 4*B*) at any tested expression level (Fig. S4). This indicates that liquid ordered “raft”-like plasma membrane phases are not relevant for the formation of mGFP–hDAT dimers.

Previous observations from us indicated that in the case of hSERT, stability and kinetic trapping at the plasma membrane is mediated by its direct binding to PIP_2_, which promotes spatial decoupling of oligomer formation and provides cells with the ability to define quaternary structures independent of expression levels (20). We anticipated that similar to hSERT, PIP_2_ would also play a role in the stability of mGFP–hDAT dimers. We were surprised to find that mGFP–hDAT dimer distribution was not altered by changes in PIP_2_ levels at the plasma membrane (Fig. 5) indicating that unlike hSERT, mGFP–hDAT forms stable dimers in a PIP_2_-independent manner. It has been reported that the N terminus of monoamine transporters acts as a lever in amphetamine-induced efflux (72) and impacts on the overall conformational equilibrium (73). Hence, it is not surprising that the direct binding of PIP_2_ to the N terminus of hDAT plays an important role in amphetamine-induced, hDAT-mediated DA efflux (74). Whether the stable quaternary equilibrium of monomeric and dimeric species, unaffected by PIP_2_ manipulation, plays a functional role in amphetamine-induced substrate efflux awaits further experimental verification.

Let us compare our results with molecular dynamics simulations of hDAT oligomerization. hDAT shows a small number of dimeric interfaces and dimer conformations, when simulated in a cholesterol-free membrane containing only palmitoyl oleoyl-phosphatidylcholine lipids revealed (16). Some of these interfaces were mutually exclusive, others were of low abundance. The dimer conformations that showed a low probability may have been too weak to be detectable by our TOCCSL approach. Also, the employed Martini coarse-grained force field was recently shown to overstabilize protein dimers in membranes (75). The largest clusters of dimers observed in the simulations can be classified into two groups. (i) Dimers, which showed a large interaction interface between transmembrane helices all across the membrane. The stability of these dimers is dominated by direct protein–protein contacts, from which it can be inferred, that a modulatory role of lipids might be minor. (ii) Dimers with interfaces, to which lipids make a large contribution and involve extra- and intracellular loops. These can be expected to be strongly dependent on lipid composition and/or membrane thickness. At this stage, it may be premature to speculate about the mechanism that stabilizes the experimentally observed hDAT dimers: whereas it appears attractive to ascribe this to the first group, one should note that the membrane composition of the computational and the present study is very different, and depletion of cholesterol as probed here, is likely not sufficient to mirror the single lipid species used in the simulation experiments. Although the nature of the attractive forces that connect the hDAT dimers is not known, high molecular weight DAT complexes were observed in synaptosomal preparations from rats pre-exposed to multiple doses of methamphetamine, which got attenuated but not abolished upon exposure to β-mercaptoethanol (76). In line with that, hDAT in the plasma membrane is natively not cross-linked, but can be cross-linked (Cys-306) to form stable dimers by the flexible cross-linker bis-EA or by CuP-generated oxidation such that the apparent molecular mass with nonreducing SDS-PAGE increased from ∼85 to ∼195 kDa (36). Hence, it may well be that disulfide bridges are also involved in the dimer formation.

In conclusion, hDAT forms stable dimers at the plasma membrane in a PIP_2_-independent manner. What leads to the stability of mGFP–hDAT dimers is a matter of further study but it would be interesting to discuss some of the possibilities. (i) mGFP–hDAT interacts with other hitherto unknown membrane components that stabilize the dimers. (ii) The dimerization process of mGFP–hDAT does not depend on membrane lipids and is solely driven by the protein–protein interaction of the two interacting monomers. Furthermore, unbiased molecular dynamics simulation studies of hDAT revealed the presence of a limited number of symmetric and asymmetric dimers that involved the scaffold domain (16). The interaction interfaces differed in shape and involved transmembrane helices with some contacts through the entire membrane, whereas other formed contacts only in one membrane leaflet.

## Experimental procedures

### DAT construct

Mutagenesis of eGFP to mGFP was performed by the mutation A206K using the QuikChange Mutagenesis Kit (Agilent) in a peGFP-C1 vector (Clontech) with the forward primer, 5′-TAC CTG AGC ACC CAG TCC AAA CTG AGC AAA GAC CCC AAC-3′, and the reverse primer, 5′-GTT GGG GTC TTT GCT CAG TTT GGA CTG GGT GCT CAG GTA-3′. A XhoI/HindIII fragment of hDAT cDNA was ligated into XhoI/HindIII*-*digested mGFP-C1 to produce mGFP-hDAT.

### Cell culture

WT CHO and hDAT expressing stable CHO cells were cultured at 37 °C and 5% CO_2_ in Dulbecco's modified Eagle's and F-12 Ham's (DMEM F-12, Sigma) medium supplemented with 10% FBS (Sigma), penicillin and streptomycin (Sigma). Cell lines are tested monthly for mycoplasma contamination using MycoAlert (Lonza).

### Generation of stable cell line

We performed viral infection to generate CHO cells stably expressing mGFP fused to the N terminus of hDAT. Briefly, we cloned mEGFP–hDAT into the viral expression vector pIB2 (gift from J. B. Huppa, Medical University Vienna) using Gibson Assembly (New England Biolabs) with 5′-ccgcggcggccgcggtccggATGGTGAGCAAGGGCGAGG-3′ as forward primer and 5′-CAATTGTTTTACGTATCTCGCTACACCTTGAGCCAGTGGCG-3′ as reverse primer. Viral supernatants were produced in HEK293 cells co-transfecting pIB2-mEGFP-hDAT and helper plasmids coding for viral proteins (G. Nolan, Stanford University) using Turbofect. 48 h post-transfection, viral supernatants were harvested and centrifuged at 1000 × *g* for 5 min at 21 °C to remove cell debris. CHO cells were then transduced in 24-well plates in the presence of 10 μg/ml of Polybrene (Sigma) for 24 h. Positive cells were selected with blasticidine (Invivogen, Toulouse, France) for 1 week and expanded.

### Transient transfections and uptake experiments

HEK293 cells were cultured in Dulbecco's modified Eagle's medium with high glucose (4.5 g/liter) and l-glutamine (584 mg/liter), supplemented with 10% fetal calf serum (FCS), 100 units/ml of penicillin, and 100 μg/ml of streptomycin. The cells were maintained at 37 °C in a humidified atmosphere of 5% CO_2_, 95% air on standard plastic cultureware. The cells were transiently transfected with 5 μg of plasmid DNA as required, using jetPRIME® as transfection reagent. Cells were seeded at 0.8 × 10^5^ cells/0.5 ml or 0.8 × 10^5^ cells/well densities into poly-d-lysine–coated 48-well plates 24 h after transfection with cDNAs of either WT–hDAT or mGFP–hDAT. Uptakes were performed as described previously (77). In brief, the cell medium was aspirated and the cells were washed once with 0.5 ml of Krebs-HEPES buffer at room temperature (23 °C; Krebs-HEPES buffer, composition: HEPES, 10 mm; NaCl, 120 mm; KCl, 3 mm; CaCl_2_, 2 mm; MgCl_2_, 2 mm; glucose, 20 mm; final pH 7.3–7.4). The cells were incubated with [^3^H]DA (200 nm) and various concentrations of unlabeled DA (range: 0.2-60 μm; final volume 0.1 ml/well) and substrate uptake was stopped after 1 min by exchange of the substrate containing buffer with 0.5 ml of ice-cold Krebs-HEPES buffer. The cells were lysed in 0.5 ml of 1% SDS, transferred to scintillation vials, and assayed for [^3^H] content by liquid scintillation counting. Nonspecific uptake was determined in the presence of 100 μm cocaine.

### Sample preparation

For all cell imaging experiments, 8-well glass bottom plates were used (Thermo Fisher Scientific, Nunc) and coated with poly-d-lysine (Sigma). Cleaned slides were incubated with 0.1 mg/ml of poly-d-lysine for 1 h at 37 °C and unbound poly-d-lysine were washed three times before use.

### Cholesterol oxidation

To oxidize the cholesterol in the plasma membrane, the stable cells were incubated with 2 units/ml of cholesterol oxidase (Invitrogen) in Hanks' balanced salt solution for 30 min at 37 °C.

### Cholesterol depletion

To deplete cholesterol from the plasma membrane, stable cells were incubated with 5 mm methyl-β-cyclodextrin (Sigma) in Hanks' balanced salt solution for 1 h at 37 °C.

### PIP_2_ depletion

For activation of phospholipase Cγ, cells were incubated for 20 min at 37 °C with 25 μm m-3M3FBS (Sigma) in Hanks' balanced salt solution, with calcium and magnesium (Sigma) supplemented with 2% FBS. m-3M3FBS remained in the imaging buffer during measurements.

### Microscopy

A 488-nm laser (SAPPHIRE HP, Coherent Inc.) was mode-cleaned using a pinhole and the illumination intensity and timing were adjusted with an acousto-optical modulator (model 1205, Isomet) using custom written software (Labview, National Instruments). The laser beam was focused onto the back-focal plane of a TIRF objective (NA 1.46, ×100 Plan APOCHROMAT, Zeiss) mounted on an inverted Zeiss Axiovert 200 microscope. The emission light was filtered using appropriate filter sets for GFP and imaged with a back-illuminated liquid nitrogen-cooled CCD camera (Micro Max 1300-PB, Roper Scientific). To restrict the excitation and photobleaching area an adjustable slit aperture (Zeiss) was used as field stop. All experiments were performed at room temperature. Imaging during all experiments was performed using an objective-type TIRF excitation with an excitation power of ∼0.6–0.8 kW/cm*^2^* (determined in epi-configuration) and stroboscopic illumination with excitation times of 5 ms.

### FRAP

To determine the mobile fraction of mGFP–hDAT, a ∼7 × 7-μm area of the bottom plasma membrane was irreversibly photobleached, and the fluorescence recovery over time was monitored (*n* >10 cells). Photobleaching and readout were performed in TIRF configuration. Data were analyzed using in-house algorithms implemented in Matlab (Mathworks). The central part of the bleached region was evaluated by integrating all counts and normalizing to the pre-bleach image. To calculate the mobile fraction of mGFP–hDAT, the resulting curve was fitted with *I*/*I*_0_ = α(1-exp(*t*/τ*_D_*)) yielding the mobile fraction α of mGFP–hDAT.

### TOCCSL

TOCCSL experiments were performed as described previously (28). A pre-bleach image was recorded, which was used for determination of the mGFP–hDAT surface density. After a *t*_pre_ = 50 ms, a confined region of the cell membrane was photobleached for *t*_bleach_ = 800 ms with a high laser power of ∼8–10 kW/cm^2^. To check for complete bleaching, a post-bleach image was recorded *t*_post_ = 40 ms after the bleach pulse. Finally, the TOCCSL image was recorded after an adjustable recovery time of *t*_recovery_ = 15,000–20,000 ms. Images were acquired at low excitation power of ∼0.6–0.8 kW/cm^2^ (all excitation intensities were determined in epiconfiguration).

### Repetitive TOCCSL experiment

For the hDAT dimer stability studies, a repetitive TOCCSL protocol was applied. One TOCCSL run every 5 min repeated over 50 min starting from the first bleach pulse was performed on the same region of each cell; following a modification of the timing protocol used earlier (28). Both bleaching and image acquisition were obtained in the TIRF mode. The TOCCSL image of each run was used for brightness analysis.

### Brightness analysis

For single-molecule brightness analysis, images were analyzed in Matlab using an in-house algorithm. The pixel counts obtained were subsequently converted to photon by subtracting the offset and multiplying with the inverse gain (as per specification of the Roper camera). Individual diffraction-limited fluorescent signals from the regions of interest were selected and fitted with a Gaussian intensity function. The fitting routine yielded the single spot brightness B, which was then used to determine the oligomeric state of mGFP–hDAT. Next, the obtained brightness values of each fluorescent spot in the TOCCSL image were plotted as a probability density function, ρ(B). The cells were extensively photobleached to obtain the brightness distribution of single mGFP molecules ρ_1_(B), such that each potential mGFP–hDAT oligomer contained only one active fluorophore. Using autoconvolution, the monomer brightness distribution was used to calculate the brightness distributions for dimers ρ_2_(B), trimers ρ_3_(B), and so on. The overall single spot brightness distribution ρ(B) was then fitted by a linear combination of ρ_1_(B), ρ_2_(B), ρ_3_(B) and so on.

(Eq. 1) $$\rho \left(B\right)=\sum_{N=1}^{\max}\alpha N\cdot \rho N\left(B\right)$$

Fitting ρ(*B*) in the above equation yielded the fractions α*_N_* of the different numbers of co-diffusing active mGFP molecules. To obtain error bars during our analysis, we followed a bootstrapping method. Briefly, randomly chosen subsamples containing 50% of the data were analyzed using Equation 1; the error bars represent the obtained standard deviation from 100 repetitions for each oligomeric size divided by √2.

### Matlab code

The Matlab Source code for TOCCSL analysis is available at https://github.com/schuetzgroup/TOCCSL_analysis.^4^

## Author contributions

A. K. D., H. H. S., and G. J. S. conceptualization; A. K. D. formal analysis; A. K. D., F. B., and K. J. investigation; A. K. D., O. K., F. B., and G. J. S. methodology; A. K. D. writing-original draft; T. S., H. H. S., and G. J. S. supervision; T. S., H. H. S., and G. J. S. writing-review and editing; H. H. S. and G. J. S. funding acquisition; G. J. S. resources; G. J. S. software; G. J. S. project administration.

## Supplementary Material

Supporting Information

## Footnotes

4 Please note that the JBC is not responsible for the long-term archiving and maintenance of this site or any other third party hosted site.

## Footnotes

3 The abbreviations used are:

hDAT: human dopamine transporter
DA: dopamine
GAT: γ-amino acid transporter
SERT: serotonin transporter
NET: norepinephrine transporter
ER: endoplasmic reticulum
mGFP: monomeric GFP
TIRF: total internal reflection fluorescence
TOCCSL: thinning out clusters while conserving stoichiometry of labeling
FRAP: fluorescence recovery after photobleaching
FBS: fetal bovine serum
m-3M3FBS: 2,4,6-trimethyl-*N*-(3-trifluoromethylphenyl)benzenesulfonamide
TMH: transmembrane helices
SLC6: solute carrier 6
PIP_2_: phosphatidylinositol 4,5-bisphosphate
CHO: Chinese hamster ovary
MβCD: methyl-β-cyclodextrin
eGFP: enhanced green fluorescent protein
hSERT: human serotonin transporter.
