# Supplementary material for: Dopamine transporter forms stable dimers in the live cell plasma membrane in a phosphatidylinositol 4,5-bisphosphate–independent manner
Source: J Biol Chem. 2019 Jan 31;294(14):5632–42. doi: 10.1074/jbc.RA118.006178 (PMC6462504; doi:10.1074/jbc.RA118.006178)
Supplement: Supporting Information [file supp_294_14_5632__index.html]

Dopamine transporter forms stable dimers in the live cell plasma membrane in a phosphatidylinositol-4,5-bisphosphate independent manner — Stable DAT dimers in the plasma membrane — Dopamine transporter forms stable dimers in the live cell plasma membrane in a phosphatidylinositol 4,5-bisphosphate–independent manner — Stable DAT dimers in the plasma membrane — Supporting Information 

# Dopamine transporter forms stable dimers in the live cell plasma membrane in a phosphatidylinositol 4,5-bisphosphate–independent manner

## Supporting Information

- Supporting Information (to be published online) - Supporting figures S1-S5
