# Supplementary material for: Dopamine transporter forms stable dimers in the live cell plasma membrane in a phosphatidylinositol 4,5-bisphosphate–independent manner
Source: J Biol Chem. 2019 Jan 31;294(14):5632–42. doi: 10.1074/jbc.RA118.006178 (PMC6462504; doi:10.1074/jbc.RA118.006178)
Supplement: Supporting Information [file supp_RA118.006178_141208_2_supp_276477_pm1y1z.pdf]

# Supporting Information

Dopamine transporter forms stable dimers in the live cell plasma membrane in a phosphatidylinositol-4,5-bisphosphate independent manner

**Anand Kant Das[1], Oliver Kudlacek[2], Florian Baumgart[1], Kathrin Jaentsch[2], Thomas Stockner[2], Harald H. Sitte[2*], Gerhard J. Schütz[1*]**

[1]Institute of Applied Physics, Vienna University of Technology, Getreidemarkt 9, A-1060, Vienna, Austria
[2]Center for Physiology and Pharmacology, Institute of Pharmacology, Medical University Vienna, Waehringerstrasse 13a, A-1090 Vienna, Austria

**Running title**: *Stable DAT dimers in the plasma membrane*

[*]To whom correspondence should be addressed: Harald H. Sitte, E-mail: harald.sitte@meduniwien.ac.at; Gerhard J. Schütz, E-mail: schuetz@iap.tuwien.ac.at

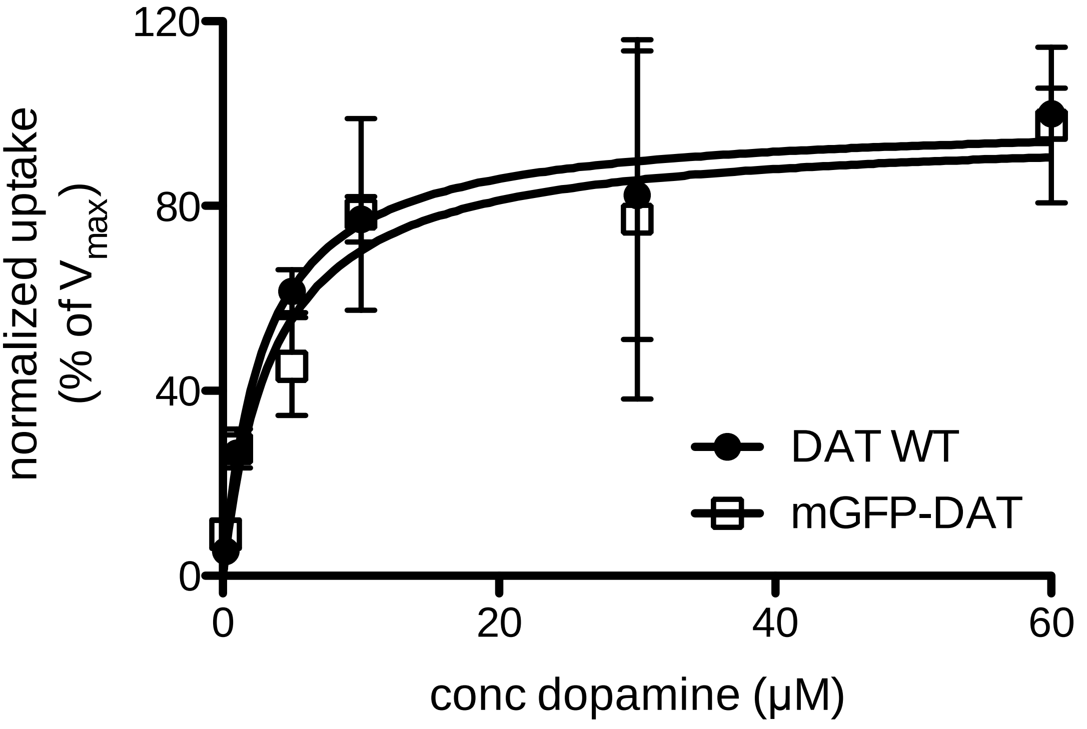


**Figure S1. [$^3$H]-DA uptake assay on wild type hDAT and mGFP-hDAT expressing cells**. Saturation uptake kinetics of [$^3$H]-DA were measured in cells transiently transfected with WT-hDAT and mGFP-hDAT. The average data were normalized by $V_{max}$ and are presented as mean ± SD (n=3). $V_{max}$ values were: WT- hDAT: 136.01 ± 44 pmol/min/mio and mGFP-hDAT: 12.74 ± 4.73 pmol/min/mio, $K_m$ values were: 2.98 ± 0.38 µM for WT- hDAT and 4.11 ± 0.85 µM for mGFP-hDAT.

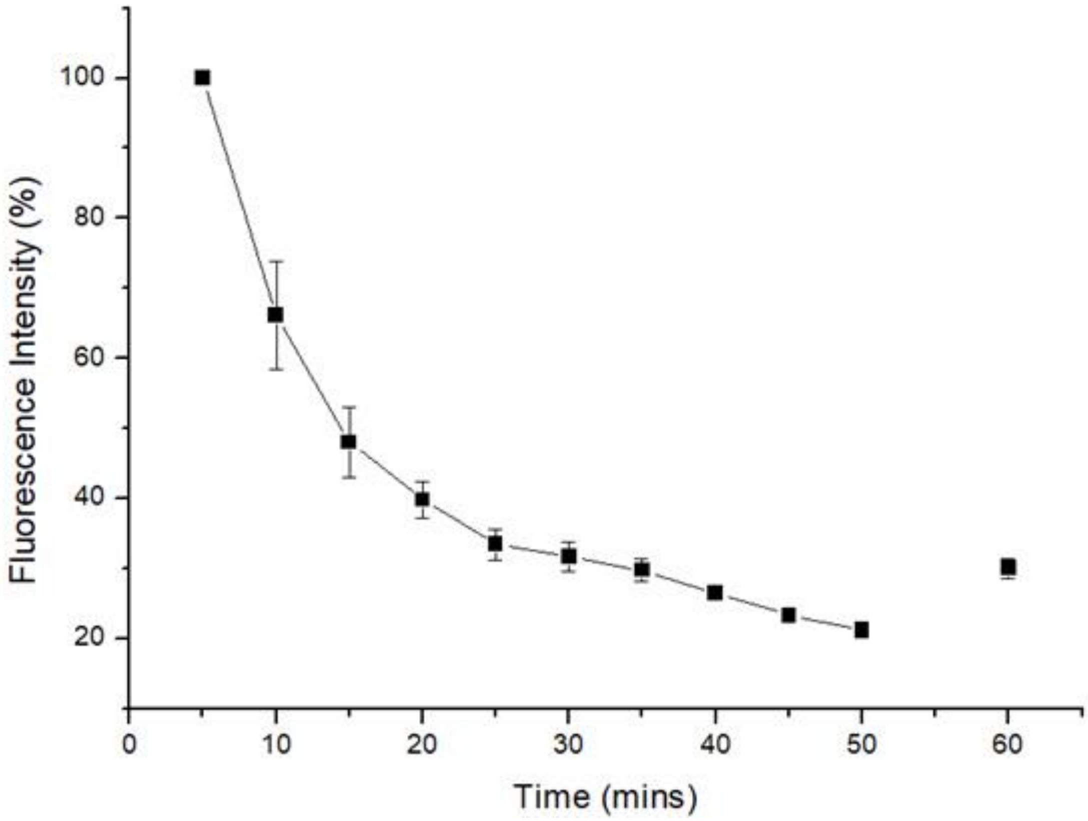


**Figure S2. Effect of repetitive TOCCSL on the fluorescence intensity at the plasma membrane.** We determined the effect of 10 repetitive TOCCSL cycles at 5 minutes interval on the overall percentage of active fluorophores at the plasma membrane. The plot shows the decrease in the brightness levels of the individual TOCCSL pre-bleach images. 10 minutes after the last TOCCSL run, we recorded the average brightness in the region of interest, which was reduced to 30% of the original value. Average data are presented ± SEM (n=5).

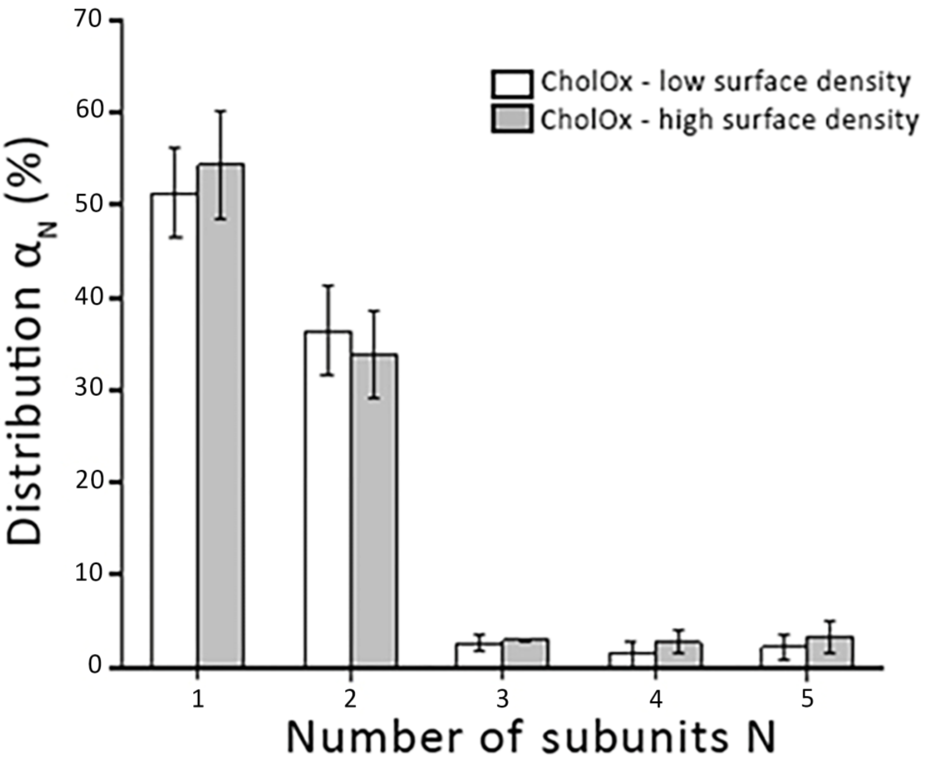


**Figure S3: Effect of cholesterol oxidation on hDAT dimer distribution at different cell densities**. The cholesterol oxidation by cholesterol oxidase has no effect on dimer distribution at low and high surface densities of mGFP-hDAT: the comparison of the two populations, high (grey) and low surface density (white), shows no difference in oligomerization. The mean density of mGFP-hDAT was evaluated by dividing the integrated fluorescent intensity of the cell membrane by the mean intensity of a monomer, yielding a mean density of ~5 mGFP-hDAT/$\mu m^2$ for low surface density and ~35 mGFP-hDAT/$\mu m^2$ for high surface density. Error bars show the s.e.m.

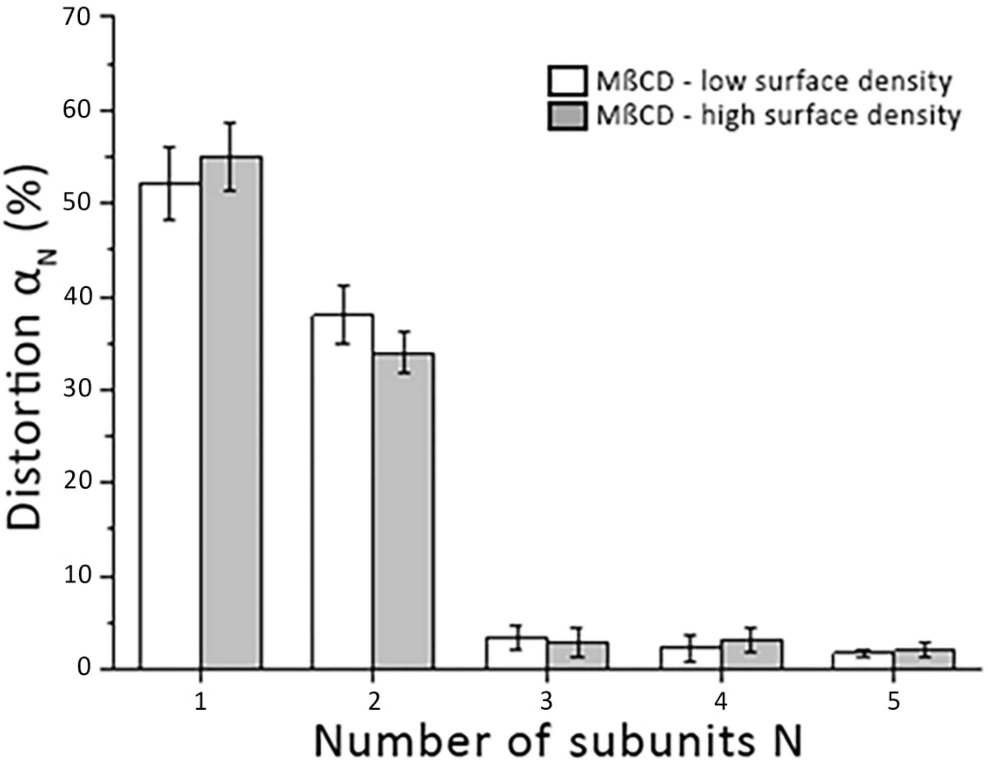


**Figure S4: Effect of cholesterol depletion on hDAT dimer distribution at different cell densities**. Depletion of cholesterol by the action of MβCD has no effect on the dimer distribution at low and high surface densities of mGFP-hDAT: the comparison of the two populations, high (grey) and low surface density (white), shows no difference in oligomerization. The mean density of mGFP-hDAT was evaluated by dividing the integrated fluorescent intensity of the cell membrane by the mean intensity of a monomer, yielding a mean density of ~6 mGFP-hDAT/$\mu m^2$ for low surface density and ~30 mGFP-hDAT/$\mu m^2$ for high surface density. Error bars show the s.e.m.

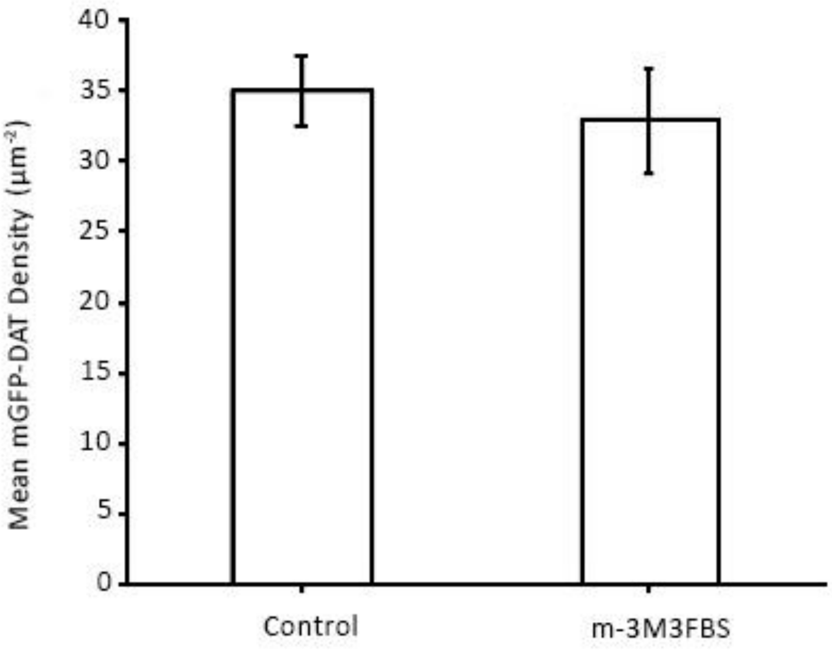


**Figure S5**: **Mean surface density of mGFP-hDAT upon $PIP_2$ depletion.** We enzymatically depleted $PIP_2$ at the plasma membrane via activation of phospholipase $C_\Upsilon$ ($PLC_\Upsilon$) by incubating cells for 20 min with the direct $PLC_\Upsilon$-activator m-3M3FBS (25 µM). The bar graph shows the mean surface density/$\mu m^2$ of untreated control cells (35 ± 3.2) and *m*-3M3FBS treated cells (33± 5.3) (n>60 cells). Error bars indicate the standard error of mean.
